# Fifty Most Cited Articles for Femoroacetabular Impingement and Hip Arthroscopy

**DOI:** 10.3389/fsurg.2015.00041

**Published:** 2015-08-18

**Authors:** Simon Lee, Jason Shin, Marc Haro, Michael Khair, Jonathan C. Riboh, Benjamin D. Kuhns, Charles A. Bush-Joseph, Shane J. Nho

**Affiliations:** ^1^Rush University Medical Center, Chicago, IL, USA; ^2^University of Saskatchewan, Saskatoon, SK, Canada; ^3^Medical University of South Carolina, Charleston, SC, USA

**Keywords:** hip arthroscopy, femoroacetabular impingement, top 50, sports surgery, citation review

## Abstract

Growing awareness of femoroacetabular impingement (FAI) and recent innovations in management have resulted in hip arthroscopy becoming one of the fastest-growing orthopedic subspecialties. The purpose of this study was to identify the 50 most cited articles related to the topic of FAI and hip arthroscopy and to analyze their characteristics. The overall number of citations within these articles ranged from 99 to 820. Citation density ranged from 4.41 to 74.55. Seven countries produced these articles with the majority attributed to the United States (*n* = 26) and Switzerland (*n* = 18). Clinical studies made up more than half of the top articles (*n* = 27). The Journal of Bone and Joint Surgery level of evidence most commonly encountered was level IV (*n* = 24), while the remaining articles were level III (*n* = 3). No randomized controlled trials or non-randomized controlled trials were encountered in this search. The level of evidence was not significantly correlated with the overall number of citations, publication year, or citation density. The current top 50 list provides orthopedic surgeons interested in hip arthroscopy with an up-to-date core list of the most cited articles in the scientific literature and represents a foundation to use to develop their knowledge regarding hip arthroscopy and FAI.

## Introduction

Analyses of “most cited articles” have been performed in the field of orthopedics as a specialty in general (1, 2), as well as in various orthopedic subspecialties, including pediatrics, hand, shoulder, arthroscopy, and total joint arthroplasty (3–7). However, no such study has been published on “hip arthroscopy” or “femoroacetabular impingement (FAI).” In the current literature, a study identifying the top 25 cited articles in orthopedic arthroscopy in 2012 by Gheiti et al. (5) included only one article related to hip joint.

While Smith-Peterson first described FAI in 1935 as the “impingement of the femoral neck on the anterior acetabular margin” in a 55-year-old male patient, the concept of FAI has only relatively recently become widely accepted as a source of hip pain and dysfunction. This is due, in large part, to the extensive work by Ganz and colleagues (8–10). Similarly, the first clinical report of hip arthroscopy was published by Takagi in 1939 (11), but it was not until the 1980s that hip arthroscopy became more widely utilized in the diagnosis and treatment of hip pathology (12). The understanding of FAI as well as the surgical technique and clinical outcomes following hip arthroscopic procedures has grown exponentially.

The purpose of this study was to identify the 50 most cited articles related to the topic of FAI and hip arthroscopy and to analyze their characteristics. By this method, we sought to create an up-to-date list of the most important articles in this emerging field.

## Methods

This study did not require approval from institutional review board as it involved publicly available data. Following previously described methods (2, 4, 6), we used the “cited reference search” through the ISI Web of Knowledge (Thompson Reuters, New York, NY, USA) to identify the top 50 cited articles in FAI and hip arthroscopy. The search was performed in October 2014. Articles published in any of the 67 journals categorized under the topic heading of “orthopedics” in the ISI Web of Science, which include general and subspecialty-specific, clinical, and basic science orthopedic journals, as well as physical therapy journals, were considered. The following search terms were used: “Hip Arthroscopy,” “Femoroacetabular Impingement,” and “Hip Labral Tears.” Although our primary aim was to include articles that arthroscopic hip surgeons would find relevant to their practice, we also chose to include articles describing open management of FAI as we felt that the history and evolution as well as alternative treatments of FAI would be of interest to an arthroscopic hip surgeon. Moreover, studies pertaining to diagnostic, perioperative, and postoperative management of FAI were included. Articles that were considered to be relevant to FAI and hip arthroscopy were reviewed by two authors and the decision to include or exclude was made through consensus. The articles were then ranked according to the number of highest citations to generate a list of 50 articles. When the articles had identical number of citations, the paper with higher citation density (defined as numbers of citations per year since publication) was ranked higher.

While reviewing the identified articles, the following information was recorded: article title, source journal of the article, first author, corresponding author, year of publication, country of origin (in accordance with the corresponding author’s address), and article type (basic science, clinical research article, and diagnostic studies). Clinical studies were further subtyped as randomized controlled trial, cohort study, case series, review article, case report, or expert opinion and assigned a level of evidence based on the Journal of Bone and Joint Surgery (JBJS) criteria (13).

All statistical analyses were calculated using SPSS statistical software (SPSS Inc., Chicago, IL, USA).

## Results

**Figure 1:**
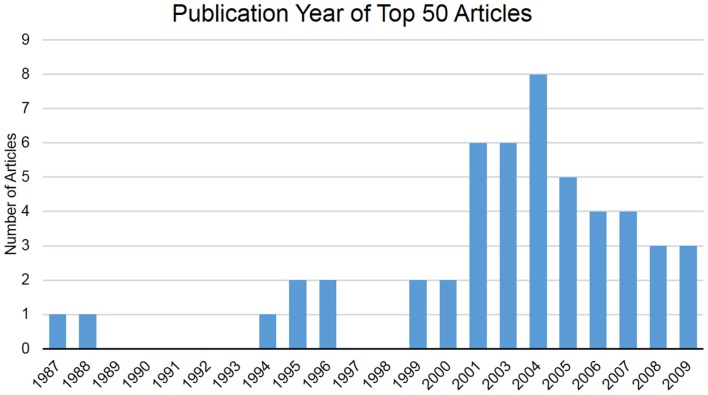
**Publication year of top 50 articles**.

There were 50 total publications included in this top FAI and hip arthroscopy articles list (Table 1). The overall number of citations within these articles ranged from 99 to 820, with an average of 182.70 ± 133.50 citations per article. Citation density for these articles ranged from 4.41 to 74.55, with an average density of 18.55 ± 13.33. The total amount of citations attributed to these articles was calculated to be 9135. These articles were published between 1987 and 2009, with 2004 producing the greatest number of top articles (*n* = 8) (Figure 1). The average number of years since publication of these articles was 11.34 ± 4.88. The selected articles were published in 10 of the 67 examined orthopedic journals, with Clinical Orthopaedics and Related Research containing the greatest amount of publications (*n* = 18) followed by Arthroscopy: The Journal of Arthroscopic and Related Surgery (*n* = 11) (Table 2). When analyzed by total citation, Clinical Orthopaedics and Related Research represented the highest citation count (*n* = 3611) followed by the JBJS-British Volume (*n* = 1600). Every article was published in the English language.

**Table 1:** **List of the top 50 cited articles in femoroacetabular impingement and hip arthroscopy with total citations and citation density**.

Rank	Article	Total citations	Citation density
1	Ganz et al. (2003). Femoroacetabular impingement: a cause for osteoarthritis of the hip (9)	820	74.55
2	Beck et al. (2005). Hip morphology influences the pattern of damage to the acetabular cartilage: femoroacetabular impingement as a cause of early osteoarthritis of the hip (14)	529	58.78
3	Ganz et al. (2001). Surgical dislocation of the adult hip a technique with full access to the femoral head and acetabulum without the risk of avascular necrosis (8)	435	33.46
4	Ito et al. (2001). Femoroacetabular impingement and the cam-effect. A MRI-based quantitative anatomical study of the femoral head-neck offset (15)	376	28.92
5	Beck et al. (2004). Anterior femoroacetabular impingement: part II. Midterm results of surgical treatment (16)	346	34.60
6	Siebenrockwt al. (2003). Anterior femoroacetabular impingement due to acetabular retroversion. Treatment with periacetabular osteotomy (17)	286	26.00
7	Ganz et al. (2008). The etiology of osteoarthritis of the hip: an integrated mechanical concept (10)	265	44.17
8	Lavigne et al. (2004). Anterior femoroacetabular impingement: part I. Techniques of joint preserving surgery (18)	239	23.90
9	McCarthy et al. (2001). The Otto E. Aufranc Award: The role of labral lesions to development of early degenerative hip disease (19)	237	18.23
10	Byrd and Jones (2000). Prospective analysis of hip arthroscopy with 2-year follow-up (20)	203	14.50
11	Espinosa et al. (2007). Treatment of femoroacetabular impingement: preliminary results of labral refixation. Surgical technique (21)	200	25.00
12	Philippon et al. (2009). Outcomes following hip arthroscopy for femoroacetabular impingement with associated chondrolabral dysfunction: minimum two-year follow-up (22)	196	39.20
13	Fitzgerald (1995). Acetabular labrum tears. Diagnosis and treatment (23)	195	10.26
14	Murphy et al. (2004). Debridement of the adult hip for femoroacetabular impingement: indications and preliminary clinical results (24)	178	17.80
15	Tanzer and Noiseux. (2004). Osseous abnormalities and early osteoarthritis: the role of hip impingement (25)	177	17.70
16	Meyer et al. (2006). Comparison of six radiographic projections to assess femoral head/neck asphericity (26)	166	20.75
17	Wenger et al. (2004). Acetabular labral tears rarely occur in the absence of bony abnormalities (27)	165	16.50
18	Siebenrock et al. (2004). Abnormal extension of the femoral head epiphysis as a cause of cam impingement (28)	154	15.40
19	Seldes et al. (2001). Anatomy, histologic features, and vascularity of the adult acetabular labrum (29)	151	11.62
20	Farjo et al. (1999). Hip arthroscopy for acetabular labral tears (30)	150	10.00
21	Myers et al. (1999). Anterior femoroacetabular impingement after periacetabular osteotomy (31)	149	9.93
22	Ikeda et al. (1988). Torn acetabular labrum in young patients. Arthroscopic diagnosis and management (32)	148	5.69
23	Philippon et al. (2007). Femoroacetabular impingement in 45 professional athletes: associated pathologies and return to sport following arthroscopic decompression (33)	143	20.43
24	Larson and Giveans (2008). Arthroscopic management of femoroacetabular impingement: early outcomes measures (34)	135	22.50
25	Peters and Erickson. (2006). Treatment of femoroacetabular impingement with surgical dislocation and débridement in young adults (35)	135	16.88
26	Kelly et al. (2005). Arthroscopic labral repair in the hip: surgical technique and review of the literature (36)	132	14.67
27	Byrd (1994). Hip arthroscopy utilizing the supine position (37)	132	6.60
28	Philippon et al. (2007). Arthroscopic management of femoroacetabular impingement: osteoplasty technique and literature review (38)	127	18.14
29	Beaulé et al. (2005). Three-dimensional computed tomography of the hip in the assessment of femoroacetabular impingement (39)	127	14.11
30	Leunig et al. (2004). Magnetic resonance arthrography of labral disorders in hips with dysplasia and impingement (40)	127	12.70
31	Wagner et al. (2003). Early osteoarthritic changes of human femoral head cartilage subsequent to femoroacetabular impingement (41)	125	11.36
32	Anderson et al. (2001). Hip and groin injuries in athletes (42)	125	9.62
33	Leunig et al. (2009). The concept of femoroacetabular impingement: current status and future perspectives (43)	124	24.80
34	Byrd and Jones (2004). Diagnostic accuracy of clinical assessment, magnetic resonance imaging, magnetic resonance arthrography, and intra-articular injection in hip arthroscopy patients (44)	124	12.40
35	Larson and Giveans (2009). Arthroscopic debridement versus refixation of the acetabular labrum associated with femoroacetabular impingement (45)	123	24.60
36	Clohisy et al. (2008). A systematic approach to the plain radiographic evaluation of the young adult hip (46)	122	20.33
37	Kelly et al. (2003). Hip arthroscopy: current indications, treatment options, and management issues (47)	122	11.09
38	Beaulé et al. (2007). Quality of life following femoral head-neck osteochondroplasty for femoroacetabular impingement (48)	121	17.29
39	Mintz et al. (2005). Magnetic resonance imaging of the hip: detection of labral and chondral abnormalities using non-contrast imaging (49)	120	13.33
40	Lage et al. (1996). The acetabular labral tear: an arthroscopic classification (50)	120	6.67
41	Glick et al. (1987). Hip arthroscopy by the lateral approach (51)	119	4.41
42	Burnett et al. (2006). Clinical presentation of patients with tears of the acetabular labrum (52)	116	14.50
43	Clarke et al. (2003). Hip arthroscopy: complications in 1054 cases (53)	115	10.45
44	Jamali et al. (2007). Anteroposterior pelvic radiographs to assess acetabular retroversion: high validity of the “cross-over-sign” (54)	113	16.14
45	Eijer et al. (2001). Anterior femoroacetabular impingement after femoral neck fractures (55)	111	8.54
46	Santori and Villar (2000). Acetabular labral tears: result of arthroscopic partial limbectomy (56)	106	7.57
47	McCarthy et al. (2003). Anatomy, pathologic features, and treatment of acetabular labral tears (57)	105	9.55
48	Byrd (1996). Labral lesions: an elusive source of hip pain case reports and literature review (58)	102	5.67
49	McCarthy and Busconi (1995). The role of hip arthroscopy in the diagnosis and treatment of hip disease (59)	100	5.26
50	Tannast et al. (2005). Tilt and rotation correction of acetabular version on pelvic radiographs (60)	99	11.00

**Table 2:** **List of the journals of the top 50 articles are published within along and the total amount of citations each journal accounts for**.

Journal	No. of top 50 articles	% of top 50 articles	Total citations
*Clinical Orthopedics and Related Research*	18	36.0	3812
*Arthroscopy*	11	22.0	1442
*Journal of Bone and Joint Surgery-American Volume*	6	12.0	980
*Journal of Bone and Joint Surgery-British Volume*	5	10.0	1684
*American Journal of Sports Medicine*	4	8.0	498
*Journal of Orthopedic Research*	2	4.0	240
*Journal of Orthopedic Trauma*	1	2.0	111
*Knee Surgery Sports Traumatology Arthroscopy*	1	2.0	143
*Orthopedics*	1	2.0	100
*Osteoarthritis and Cartilage*	1	2.0	125

Seven countries produced these top articles with the vast majority being attributes to the United States (*n* = 26) and Switzerland (*n* = 18). Canada (*n* = 2), Brazil (*n* = 1), Italy (*n* = 1), Japan (*n* = 1), and the United Kingdom (*n* = 1) did not produce more than two articles each. While the United States represented the highest amount of articles in the top 50, the articles originating from Switzerland contained a greater amount of total citations (US: 3684, Switzerland: 4664), a higher citation average per article (US: 141.69, Switzerland: 259.11), higher total citation density (US: 382.19, Switzerland: 480.00), and a greater average citation density (US: 14.70, Switzerland: 26.67).

Ganz had the highest cited article with his 2003 publication “Femoroacetabular impingement – a cause for osteoarthritis of the hip.” JWT Byrd held the most first-authored articles with 4 of the top 50, closely followed by Ganz, McCarthy, and Philippon with 3 first-authored articles each. However, when this list was analyzed by corresponding authors, Leunig was listed as the corresponding author for the most with 5, followed by Ganz and Byrd with 4, and Philippon and McCarthy with 3.

Stratifying the top 50 articles by citation density, Ganz again continued to represent the top article (74.55 citations per year), while Beck had the second densest article (58.78 citations per year). Dr. Ganz had the third most densely cited article as well (44.17). In fact, Dr. Ganz published three of the most densely cited articles within the top 10 and Beck had two of the most densely cited articles in the same list.

Clinical studies made up more than half of the top articles (*n* = 27), while the remaining articles comprised review type articles (*n* = 12), radiographic and/or diagnostic based articles (*n* = 7), and basic science articles (*n* = 4). Among the clinically based articles, the JBJS level of evidence most commonly encountered was level IV (*n* = 24), while the remaining articles were level III (*n* = 3). No randomized controlled trials or non-randomized controlled trials were encountered in this search. The level of evidence was not significantly correlated with the overall number of citations (*R* = 0.043, *P* = 0.767), publication year (*R* = 0.211, *P* = 0.142), or citation density (*R* = −0.014, *P* = 0.924).

The most recently published article on the top 50 list was published in 2009 by Leunig et al., and while it ranks 33rd in total citations (124 total citations), this article ranked 11th in terms of citation density. The earliest article was published in 1987 by Glick, ranking 41st in terms of total citations (119 total citations); however, it was the least densely cited article among the top 50 papers (4.41). Interestingly, while the year of publication does not significantly correlate with total citations, it does so with citation density (*R* = 0.023, *P* = 0.002). There were no significant differences when comparing clinical basic science type studies in relation to total citations or citation density (*P* > 0.05).

## Discussion

Femoroacetabular impingement is a relatively new concept when compared to other classically described pathologies in the field of orthopedic surgery. However, growing awareness of this condition and its management as well as recent technical advancements have resulted in arthroscopic hip surgery becoming one of the fastest-growing orthopedic subspecialties. The rapid development of this field has been closely correlated with increasing interest in the subspecialty as well as the amount of new hip arthroscopists entering practice. Therefore, we believe that it is important to identify and analyze the most important articles in the field of hip arthroscopy in order to provide the orthopedic community with a list of essential publications critical to this emerging field. This list represents a basis of fundamental knowledge for hip arthroscopy and can act as a starting point for residency and fellowship programs aiming to train future hip arthroscopists. Although the presented articles are relatively contemporary as compared to other classic orthopedic publications, these articles were written during the development of the essential knowledge of FAI and the field of hip arthroscopy. Therefore, they represent the field’s scientific foundation and are valuable for clinicians who are developing their own management techniques.

We analyzed the characteristics of these articles to determine what qualities make an orthopedic article important to authors writing about FAI and hip arthroscopy. We found that the majority of articles where either published from the United States or Switzerland. Other reviews of classic articles in general surgery, plastic surgery, anesthesia, emergency medicine, and orthopedic surgery have similarly found a predominance of American authors within classic literature (1, 2, 61–64). However, this current review found that the number of articles originating from the United States when compared to other countries is proportionally lower as compared to the previously referenced specialties. Switzerland in particular represents a high proportion of top articles within the classic FAI and hip arthroscopy literature. This is not surprising as several of the clinicians who initially and extensively studied FAI in hip arthroscopy originate from Switzerland.

We found that the majority of published articles were clinical studies; however, these papers were primarily limited to level IV and level III evidence. Given the relative infancy of the field, such level of evidence is not unexpected. However, we do not believe that this lower level of evidence detracts from the value or importance of these articles. We expect that as the quantity of practitioners and patients continues to grow with time, additional studies with increasing patient cohort sizes and possibly higher levels of evidence will be conducted and published. The recent emphasis placed on “evidence-based medicine” in clinical practice encourages high-quality research to provide a basis for clinical management paradigm, and current and future studies will draw from classic literature presented in this list to accomplish this goal.

In addition to the clinical studies, we also found that a significant number of the top 50 articles were categorized as basic science studies. Many of these articles were biomechanical studies utilizing cadaveric tissue to elucidate the potential pathophysiology of symptomatic hip pain related to FAI or to develop novel surgical techniques. The remaining articles were imaging-based in nature and sought to improve the ability of clinicians to appropriately diagnose and evaluate pathological FAI lesions, as well as allow increased precision in preoperative planning. As we develop new surgical techniques, improve preoperative planning, and optimize postoperative rehabilitation, these articles provide the basis of knowledge necessary to advance these areas. Additionally, a minority of the articles found in this list were review type publications. As the current scientific literature for FAI and hip arthroscopy is still developing, the process of understanding this disease process and surgical technique is ongoing. As the scientific literature continues to mature in volume as well as quality, more comprehensive reviews will be possible, providing valuable summaries for the aspiring clinician.

Although the total amount of citations attributed to articles listed in the current top 50 list is lower as compared to the top 100 articles in orthopedic surgery and even when compared to the top 50 articles in shoulder surgery, we found that the citation density of top FAI and hip arthroscopy articles was generally greater. For example, Lefaivre et al. identified “Traumatic arthritis of the hip after dislocation and acetabular fractures: treatment by mold arthroplasty. An end-result study using a new method of result evaluation” by Harris as the top-cited article in orthopedic surgery with 1748 total citations; however, the citation density of this article was only 41.62 (2). Similarly, Namdari et al. identified “A clinical method of functional assessment of the shoulder” by Constant et al. as the top-cited article in shoulder-specific orthopedic surgery with 1211 total citations, but this paper only had a citation density of 50 (4). The top-cited article in the current study by Ganz had a total of 820 citations, significantly lower than the top orthopedic surgery or shoulder specific articles, but its citation density was higher at 74.55. We found this to be a trend within the current top 50 articles list when compared to other top-cited article publications in the scientific literature. As citation density may be used as a proxy of current interest in any particular field or article, the current study demonstrates that FAI and hip arthroscopy are emerging and impactful topics in the current orthopedic scientific community. Therefore, while the articles identified within this analysis are significantly more contemporary as compared to other “classic” orthopedic concepts, the high level of activity within the subspecialty may benefit from this list.

## Limitations

This study has limitations. All previously established methods of analyzing and ranking the top articles within the specific medical field contain limitations in their evaluation processes, including those directed toward orthopedic surgery. Similar to previously described methods, our analysis did not account for self-citations, oral or poster presentations, textbooks citations, as well as the intrinsic bias of citing articles from the Journal of which they intend to publish. Self-citation in particular may be particularly important as high-volume authors may have a tendency to cite their own previous articles in their newly submitted work. An additional limitation of our methodology is that the categorization of journals and citations in web of science may lead to the omission of influential articles about FAI and hip arthroscopy from known-orthopedic journals. However, our aim was to provide orthopedic surgeons list of the top 50 most impactful articles on a specific subspecialty as opposed to orthopedic surgery in general, so therefore we believe that the vast majority of articles on FAI and hip arthroscopy will be found within these 61 analyzed orthopedic journals and that these publications truly reflect the most important works in this subspecialty. An additional weakness attributed to this methodology is that work published prior to 1945 could not be included within the initial search as the citation database was non-existent at that time. The impact of this, however, was probably minimal within our research for articles about FAI and hip arthroscopy, since these concepts are relatively recent and have only been established in the scientific literature within the past three decades. In fact, while the initial article that we encountered within the top 50 list was published in 1987, the vast majority of the remaining articles were produced in the 2000s.

Finally, as this analysis is a cross-sectional study at one point in time, we can only develop conclusions based on the citation counts of these articles at that particular time. As new developments and scientific knowledge and techniques continue to evolve within the subspecialty, major paradigm shifts in management based on more relevant articles may significantly altered this list in the future. In this situation, it would be prudent to establish the validity of this list at a later time as the field continues to mature.

## Conclusion

We rank the top 50 articles in the subspecialty of arthroscopic hip surgery and FAI. We found that these articles written in English, most commonly published in *Clinical Orthopaedics and Related Research*, were primarily level III and level IV observational clinical studies, but with a significant amount of basic science research as well. The current top 50 list provides orthopedic surgeons interested in hip arthroscopy with a list of the most important articles in the scientific literature currently and represents a foundation that young clinicians can use to develop their knowledge regarding hip arthroscopy and FAI.

## Conflict of Interest Statement

Shane J. Nho is a paid consultant for Stryker, Pivot Medical, and Ossur; owns stock in Pivot Medical; and receives research support from Arthrex, Linvatec, Smith and Nephew, DJ Orthopaedics, Miomed, Athletico, Stryker, Pivot Medicine, and Allosource. Charles Bush-Joseph is an unpaid consultant for The Foundry and is on the Medical Publications editorial/governing board for the American Journal of Sports Medicine. The remaining authors declare that the research was conducted in the absence of any commercial or financial relationships that could be construed as a potential conflict of interest.
